# Clinical Applications and Factors Involved in Validating Thermal Windows Used in Infrared Thermography in Cattle and River Buffalo to Assess Health and Productivity

**DOI:** 10.3390/ani11082247

**Published:** 2021-07-30

**Authors:** Daniel Mota-Rojas, Alfredo M. F. Pereira, Dehua Wang, Julio Martínez-Burnes, Marcelo Ghezzi, Ismael Hernández-Avalos, Pamela Lendez, Patricia Mora-Medina, Alejandro Casas, Adriana Olmos-Hernández, Adriana Domínguez, Aldo Bertoni, Ana de Mira Geraldo

**Affiliations:** 1 Neurophysiology, Behavior and Animal Welfare Assessment, DPAA, Universidad Autónoma Metropolitana (UAM), Unidad Xochimilco, Mexico City 04960, Mexico; ale0164g@hotmail.com (A.C.); mvz.freena@gmail.com (A.D.); aldo_bm@hotmail.com (A.B.); 2 Mediterranean Institute for Agriculture, Environment and Development (MED), Institute for Advanced Studies and Research, Universidade de Évora, Pólo da Mitra, Ap. 94, 7006-554 Évora, Portugal; apereira@uevora.pt; 3 School of Life Sciences, Shandong University, Qingdao 266237, China; dehuawang@sdu.edu.cn; 4 Animal Health Group, Facultad de Medicina Veterinaria y Zootecnia, Universidad Autónoma de Tamaulipas, Victoria City 87000, Mexico; jmburnes@docentes.uat.edu.mx; 5 Animal Welfare Area, Faculty of Veterinary Sciences (FCV), Universidad Nacional del Centro de la Provincia de Buenos Aires (UNCPBA), Buenos Aires 7000, Argentina; ghezzi@vet.unicen.edu.ar (M.G.); palendez@vet.unicen.edu.ar (P.L.); 6 Facultad de Estudios Superiores Cuautitlán, Universidad Nacional Autónoma de México (UNAM), Cuautitlan Izcalli 54714, Mexico; mvziha@hotmail.com (I.H.-A.); mormed2001@yahoo.com.mx (P.M.-M.); 7 Division of Biotechnology—Bioterio and Experimental Surgery, Instituto Nacional de Rehabilitación-Luis Guillermo Ibarra (INR-LGII), Tlalpan, Mexico City 14389, Mexico; adrianaolmos05@yahoo.com.mx

**Keywords:** animal welfare, *Bubalus bubalis*, cows, infrared thermography, thermal window, river buffalo

## Abstract

**Simple Summary:**

Infrared thermography is a clinically useful method for detecting physiopathological alterations in animals through microvascular changes. It has been adapted for applications with large species, as a support tool in evaluating animal welfare, and can also contribute to productive and reproductive evaluations. This review discusses the thermal windows currently in use and characterizes their differences and limitations as resources for early detection and diagnosis in cattle and river buffaloes.

**Abstract:**

Infrared thermography (IRT) is a non-ionizing, non-invasive technique that permits evaluating the comfort levels of animals, a topic of concern due to the growing interest in determining the state of health and welfare of production animals. The operating principle of IRT is detecting the heat irradiated in anatomical regions characterized by a high density of near-surface blood vessels that can regulate temperature gain or loss from/to the environment by modifying blood flow. This is essential for understanding the various vascular thermoregulation mechanisms of different species, such as rodents and ruminants’ tails. The usefulness of ocular, nasal, and vulvar thermal windows in the orbital (*regio orbitalis*), nasal (*regio nasalis*), and urogenital (*regio urogenitalis*) regions, respectively, has been demonstrated in cattle. However, recent evidence for the river buffalo has detected discrepancies in the data gathered from distinct thermal regions in these large ruminants, suggesting a limited sensitivity and specificity when used with this species due to various factors: the presence of hair, ambient temperature, and anatomical features, such as skin thickness and variations in blood supplies to different regions. In this review, a literature search was conducted in Scopus, Web of Science, ScienceDirect, and PubMed, using keyword combinations that included “infrared thermography”, “water buffalo”, “river buffalo” “thermoregulation”, “microvascular changes”, “lacrimal caruncle”, “udder”, “mastitis”, and “nostril”. We discuss recent findings on four thermal windows—the orbital and nasal regions, mammary gland in the udder region (*regio uberis*), and vulvar in the urogenital region (*regio urogenitalis*)—to elucidate the factors that modulate and intervene in validating thermal windows and interpreting the information they provide, as it relates to the clinical usefulness of IRT for cattle (*Bos*) and the river buffalo (*Bubalus bubalis*).

## 1. Introduction

Infrared thermography (IRT) is a tool that is being used increasingly with farm animals due to society’s growing interest in animal welfare [1,2,3,4]. This technique detects the heat irradiated by a surface, decoding it into a temperature by a biological body, and interpreting its relation to the state of animal comfort [5]. In terms of both physiological and practical mechanisms, the principal mechanism involved in heat gain or loss is the regulation of the diameter of near-surface blood vessels; that is, the cutaneous vasodilatation that occurs in anatomical regions to permit thermal exchanges with the environment [6,7,8,9,10,11]. These regions, known as thermal windows [12,13,14,15,16], are characterized by a dense network of blood vessels, the presence of plexus, arteriovenous anastomosis, and the absence of hair [7,8]. Various authors sustain that these regions permit evaluating the state of health of an animal in a non-stressful manner [17,18,19,20,21]. Besides being a non-invasive technique, it is easy to apply and, in some cases, more economical than conventional methods (e.g., predicting residual food consumption) [22,23].

From a physiological perspective, changes in blood flow are essential because they permit thermal exchange between an animal’s skin and the environment through vasoconstriction and vasodilatation controlled by the sympathetic noradrenergic vasomotor response of the smooth muscles [24]. Adopting this approach has improved our understanding of the thermoregulation strategies characteristic of different species under cold conditions, such as reduction in the size of the tail and ears in some animals, the tails of laboratory rats, or the digital pads (*torus digitalis*) of cats, all of which function as thermal windows that can dissipate heat [9,20,25,26,27]. However, analyses conducted in this field led us to question the usefulness and viability of the thermal windows currently suggested for large ruminants, since certain anatomical aspects—hair, skin color or the lack of it, and skin thickness—can affect specific thermal windows, making them unviable in these species, though they are effective in others [21,28]. These factors also impede validating these windows, as has been achieved with others [29]. Nevertheless, the evidence available signals the following regions as potential thermal windows for cattle and river buffaloes: the lacrimal caruncle of the eye in the orbital region (*regio orbitalis*), the muzzle and external nose of the nasal region (*regio nasalis*), the mammary gland of the udder region (*regio uberis*), and the vulva of the urogenital region (*regio urogenitalis*) [30,31].

Choosing a thermal window that is sensitive and specific is of vital importance for evaluating the physiological state of animals, while simultaneously minimizing external influences on results [32]. This review discusses recent scientific findings on the thermal windows of the orbital (*regio orbitalis*), nasal (*regio nasalis*), udder (*regio uberis*), and urogenital regions (*regio urogenitalis*) to elucidate the individual and environmental factors that intervene in the validation and interpretation of these windows and their clinical usefulness for evaluating thermal comfort and welfare in two species: cattle (*Bos taurus* and *Bos indicus*) and river buffaloes (*Bubalus bubalis*).

## 2. Anatomical Locations of Thermal Windows in Cattle and River Buffalo

IRT is a non-invasive, non-ionizing technique that makes it possible to evaluate the thermal state of a biological body by detecting changes in the long wave radiation (infrared) emitted by specific anatomical regions [32,33]. The main characteristics that these topographical zones require are a high density of near-surface blood vessels, the absence of hair or fur, and arteriovenous anastomosis. These conditions exist, for example, in the eye of the orbital region (*regio orbitalis*) [34,35], the auricular pavilion of the ear in the auricular region (*regio auricularis*), or are traversed by large, straight vessels, as in the tail region (*regio caudalis*) of rats. Regions that satisfy these requirements are called thermal windows [16,30,36,37]. The high density of blood vessels near the dermal surface is critical because modifications of their diameter affect blood flow and change the heat exchange rate in the zone [38]. Vasodilatation permits the increase of sensible heat loss, while vasoconstriction exerts the opposite effect through control of the vasomotor response of the sympathetic adrenergic fibers in the smooth muscle of the blood vessels and in the pallidus nucleus of the rostral raphe of the spinal cord, which regulate the systemic thermoregulation process [24,39].

Studies have described that the medullary raphe participates significantly in controlling vasomotor activity, since it contains GABAergic neurons (gamma aminobutyric acid) that permit blood flow near the cutaneous surface through vasoconstriction and vasodilatation of blood vessels [24]. In experimental studies, the above has been demonstrated where suppressing these neurons with GABAergic receptor antagonists inhibited the vasodilatation capacity in rat tail regions (*regio caudalis*) and rabbit ears in the auricular region (*regio auricularis*) [40,41].

Another aspect of underscoring is the disposition of adipose tissue, which also performs a critical function in thermoregulation, as in the interscapular regions of newborn lambs and mice. In this case, during exposure to cold climatic conditions, vascular density increases significantly and activates angiogenesis that, in turn, causes an increase in metabolic activity in the zone in the form of a non-shivering thermogenic response [26,42].

**Figure 1:**
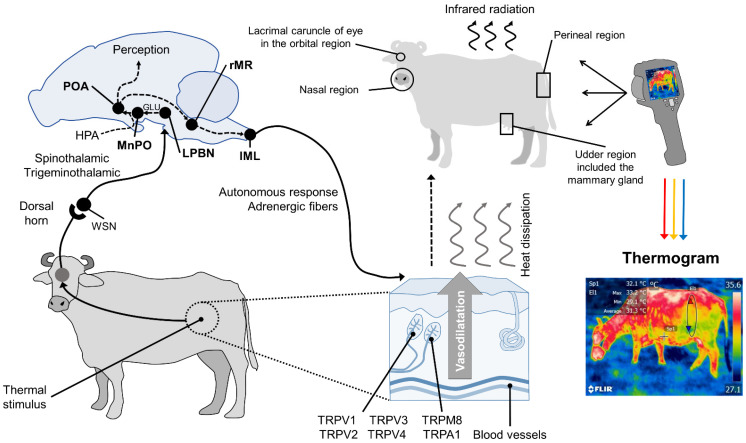
Hypothalamic neuromodulation of thermoregulation and its cutaneous response. In mammals, the thermoregulatory response begins on the periphery with thermoreceptors in the dermis, mostly heat-activated transient receptor potential vanilloids (TRPV, TRPV1, TRPV2, TRPV3, TRPV4), or cold receptors, called melastatin-related transient receptor potentials (TRPM8, TRPA1). These receive afferent information from a thermal stimulus and send the signal to the laminae of the dorsal horn of the spinal cord. The heat-sensitive (WSN) spinothalamic and trigeminothalamic neurons in this zone relay the impulse to third-order neurons in brain structures, such as the lateral parabrachial nucleus (LPBN). From there, they are projected by glutamatergic influence (GLU), to the median preoptic nucleus (MnPO) in the preoptic area (POA) of the hypothalamus. The hypothalamic network is responsible for integrating behavioral, neuroendocrine (mediated by the hypothalamic–pituitary–adrenal axis (HPA)), and autonomic thermoregulatory effector responses. Autonomic action, mediated by sympathetic adrenergic ganglia that receive information from the rostral medullary raphe (rMR) and lateral intermediolateral nucleus of the spinal cord (IML), induces cutaneous vasodilatation with the consequent dissipation of heat in the form of infrared radiation through certain body regions, known as thermal windows (e.g., the lacrimal caruncle of the eye in the orbital region (*regio orbitalis*), the muzzle in the nasal region (*region nasalis*), the mammary gland in the udder region (*regio uberis*), and the vulva of the urogenital region (*regio urogenitalis*). Infrared thermographic cameras can capture the radiation emitted through the skin using a color code that makes it possible to determine the minimum, mean, and maximum temperatures of the thermal window evaluated.

In summary, in response to significant changes in environmental temperatures, thermoreceptors in the skin begin to transmit towards the medullary raphe and median preoptic nucleus of the hypothalamus (MnPO), where the efferent signaling of sympathetic neurons exerts a vasomotor action by changing the diameter of blood vessels. Thermoreceptors perceive the thermal sensation, made up of ionic channels called transient potential receptors (TRP), which detect different temperature ranges. This, in turn, permits regulating thermal radiation on the surface [43]. This phenomenon is presented in Figure 1.

From a comparative perspective, however, differences exist among the anatomical sites involved in the thermal exchange. Recognition of these differences has led to an enhanced understanding of the vascular mechanisms involved in thermoregulation [25]. One example is the distribution of glands in interdigital spaces in dogs and cats that function to dissipate heat, while in ruminants and laboratory rats, the reduction of tail size under cold environmental conditions is important [16,20,26,27].

**Figure 2:**
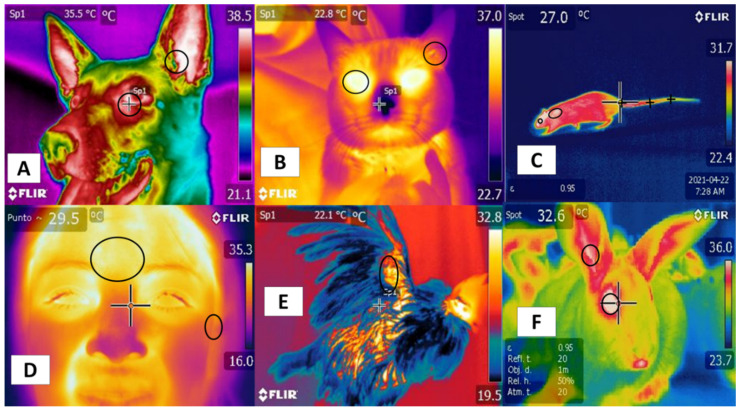
Thermal windows used in different species. (**A**) in dogs, the lacrimal caruncle in the orbital region (*regio orbitalis*) and the auricular pavilion of the ear in the auricular region (*regio auricularis*); (**B**) in cats, the eye in the orbital region (*regio orbitalis*) and the auricular pavilion of the ear in the auricular region (*regio auricularis*); (**C**) in rats, the orbital region (*regio orbitalis*), the interscapular region, and the base of the tail in the tail region; (**D**) in humans, the frontal region and the auricular pavilion of the ear in the auricular region (*regio auricularis*); (**E**) in hens, the radial region; (**F**) in rabbits, the orbital region (*regio orbitalis*) and the auricular pavilion of the ear in the auricular region (*regio auricularis*). Identification of different thermal windows has been achieved for most species.

Studies mention similarities using certain regions—such as the tails in rodents and bovines—that positively correlate with body temperature. In this region, in both the rat and the bovine tail, large straight vessels aid to the thermoregulation [20], and validating thermal windows requires high sensitivity and specificity to predict physiological states accurately. Although this has been achieved in several species [44], the reliability of this approach is limited by several conditions, such as the presence or absence of hair (glabrous skin), fur thickness, skin thickness, and fur color, all of which can alter heat gain or loss [45]. In addition, these characteristics establish differences among species, such as those between dogs and rats. In the latter, the plantar window helps evaluate thermal states [46], but it has not been possible to validate this window in dogs [47] (Figure 2). These findings indicate the need to improve our understanding of different thermal windows in light of recent scientific findings and to analyze their possible clinical usefulness for work with large ruminants. These are the topics discussed in the following sections.

Their positive correlation with the autonomic nervous system (ANS) activity, specifically the sympathetic nervous system (SNSi), has been achieved due to its activation, causing vasoconstriction of blood capillaries. However, this fact has been verified in different regions, such as the orbital (*regio orbitalis*) and auricular regions (*regio auricularis*) in dogs and cats, while in the human frontal region, it is possible to confirm this activation. In rats, rabbits, and birds, the tail (*region caudalis*) and orbital regions (*region orbitalis*) and the radial base in the antebrachial region (*regio antebrachii*), respectively, have been observed as regions that present a positive correlation with body temperature and that can provide windows to assess temperature in a non-invasive way.

## 3. Orbital Region (*Regio Orbitalis*)

**Figure 3:**
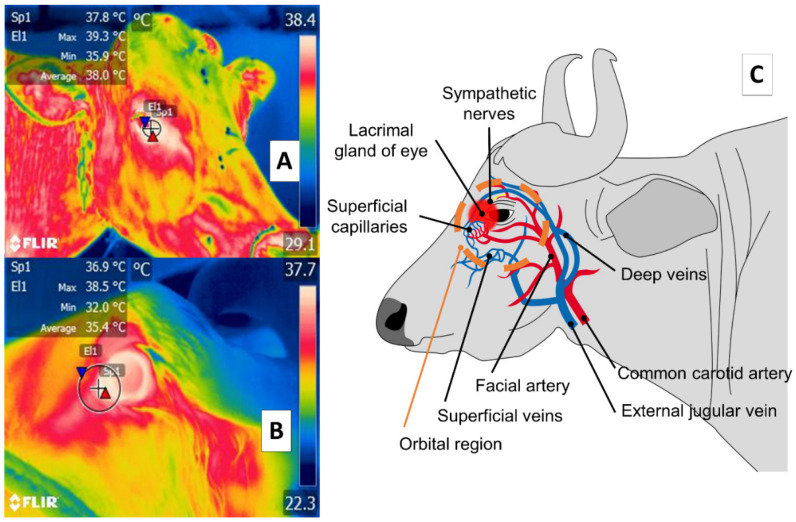
Ocular thermal window, or lacrimal caruncle of the eye in the orbital region (*region orbitalis*) (**A**) cattle (*Bos*); (**B**) river buffalo (*Bubalus bubalis*). This thermal window is outlined by a circle or square traced from the medial region of the eye 3 or 4 mm towards the rostral area of the medial palpebral commissure or canthus in the central portion of the circle around the lacrimal gland. This area is characterized by a high density of capillaries of the maxillary and infraorbital arteries that are innervated by sympathetic fibers. When stimulated, these fibers cause neurosecretion of epinephrine and norepinephrine that triggers vasoconstriction and a consequent decrease in the heat exchange rate, as occurs under conditions of stress or nociception, as shown in part (**C**) of the figure.

Some studies of large ruminants describe the lacrimal caruncle or ocular surface in the orbital region (*regio orbitalis*) as a sensitive thermal window due to its characteristic high vascularization. This is the main advantage of this region; however, disadvantages have also been mentioned, especially its susceptibility to environmental factors such as wind, direct solar radiation, and humidity, all of which can affect evaluations [48] and limit the validity of considering this zone. This region has sympathetic fibers from the facial nerve innervating the capillaries from the facial and infraorbital arteries. This peripheral vascular and nervous network is known to respond to stressful or harmful stimuli. It is represented in Figure 3, showing the location of the orbital region in the medial canthus of the eyelids [30,49,50,51]. These fibers are sensitive to neurosecretion of epinephrine and norepinephrine that promotes vasoconstriction in the capillaries, thus reducing heat exchange rate and functioning as a local thermoregulating mechanism [14]. The relation of the autonomic nervous system activity (ANS) to fluctuations in the temperature of the orbital region provides the thermal characteristics of this window [52,53].

Stewart et al. [53] evaluated pain detection during the dehorning. The study was conducted with 46 calves, 6-week-old Holstein-Friesian. There were six treatments (1) control; (2) hot-iron dehorning; (3) local anesthetic and dehorning; (4) local anesthetic control; (5) local anesthetic and nonsteroidal anti-inflammatory drug control; (6) local anesthetic, a nonsteroidal anti-inflammatory drug, and dehorning. Their study identified surface temperature variations in the lacrimal caruncle of the eye and measured heart rate variability. They observed that both the temperature of the orbital region and heart rate (HR) increased significantly during dehorning compared to basal responses. The temperature of the lacrimal caruncle of the eye, however, decreased 5 min after the procedure, while in the animals treated with ACTH, no response was generated, suggesting that action at this level clearly presented synchrony with sympathetic nerve activity (SNA)

Similar findings were described in a later study of 30, 4-week-old bull calves subjected to castration and divided into three experimental groups (one that received a local anesthetic, one without anesthesia, and a control). That study analyzed physiological parameters (HR and heart rate variability), the temperature of the lacrimal caruncle of the eye, and catecholamine levels. Results for all three groups showed a significant increase in HR, heart rate variability, and the temperature of the lacrimal caruncle of the eye during the surgical procedure compared to basal parameters. Moreover, there was a synchronic response of norepinephrine and epinephrine with the other variables. In addition, in the non-anesthetized animals, a significant reduction of the temperature of the lacrimal caruncle of the eye of 1 ± 0.5 °C was reported 10 min after surgery, compared to the other study groups [54]. In another study, monitoring the temperature of the ocular globe in the orbital region in 23 primiparous bovines (Canchim breed) for six months was an accurate means of estimating internal body temperatures and evaluating the physiological state of animals [55].

**Figure 4:**
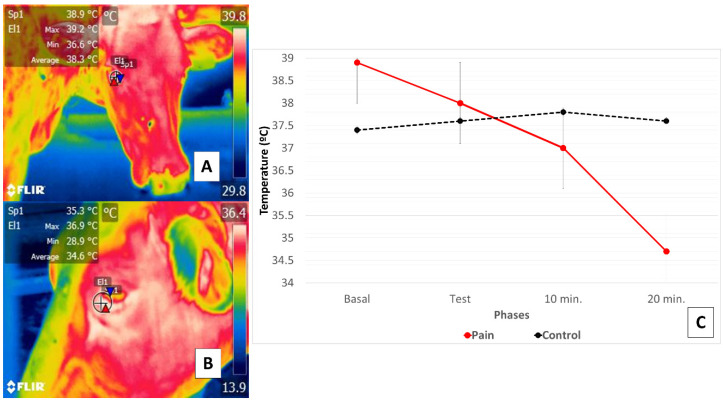
Comparison of changes in the thermal response of the lacrimal caruncle of the eye in cattle (Holstein cow) during perception of pain caused by claudication due to laminitis. This study compared the thermal response of the lacrimal caruncle of the eye in four healthy bovines under conditions of pain produced by second-degree claudication of a pelvic limb [54]. The animals were subjected to a Sprecher test [57] to assess gait. (**C**) shows that in the animals with claudication, basal temperature recordings (**A**) began at 38.9 °C, followed by a progressive decrease of 0.9 °C during testing maintained at 10- and 20-min post-test, when a total reduction of 3.3 °C was registered (**B**). Results for the healthy animals, in contrast, showed that basal temperature began at 37.4 °C but increased during testing and at 10 post-test by 0.4 °C, followed by a decrease of 0.2 °C. Thus, the phenomenon observed in the animals with claudication was attributed to perceptions of pain that provoked greater hemodynamic reactivity mediated by the neurosecretion of catecholamines that caused the surface capillaries vasoconstriction. This physiological response was mirrored in the thermal response of the lacrimal caruncle of the eye by the decrease in thermal exchange observed there.

This research supports the existence of a relation between a thermal response at the ocular level in the orbital region (*regio orbitalis*) and SNA action through activation of the sympathetic part of the autonomic nervous system (SyNS), which induces neurosecretion of catecholamines under conditions of pain and stress. Furthermore, activation of the SyNS induces peripheral vasoconstriction that reduces surface temperatures, as seen in the bovine model using the lacrimal caruncle of the eye [54,56]. The authors of the present review article sought to demonstrate this phenomenon in a preliminary study represented in Figure 4.

In this line of research, Lowe et al. [58] adapted IRT to automated calf-feeder systems on cattle-raising operations to improve productivity and animal welfare. In a study of 120 calves, temperatures of the cheek and orbital region (*regio orbitalis*) were determined using automated and conventional methods. However, both techniques and both facial regions (*regiones* *faciei*) achieved strong levels of agreement (eye: r^2^ = 0.99, *p* < 0.001; cheek at 3 × 3 pixels and 9 × 9 pixels: r^2^ = 0.85, *p* < 0.001 and r^2^ = 0.90, *p* < 0.001, respectively) that were higher for the ocular surface in the orbital region due to variability in the specific cheek regions analyzed. The importance of this study is that through continuous infrared monitoring of the animals, IRT can function as a tool for the early detection of pathological, painful, and emotional states by more readily distinguishing alterations in the temperature of thermal windows. Studies of the river buffalo mention that the ocular window can be used as an indicator of thermal comfort [21,30,31]. In this context, Chikkagoudara et al. [59] found that the temperature of the lacrimal caruncle of the eye increased by as much as 5 °C compared to a control in twenty-four 16–18 months age river buffaloes with an average weight of 301 ± 8.24 kg during experimental conditions designed to induce thermal stress similar to that of hot, dry summer days.

In contrast, Scoley et al. [60] conducted studies with 16 male and female dairy Holstein calves aged 17 days. They compared radiometric thermal images of the orbital (*regio orbitalis*) and anal (*regio analis*) regions to rectal temperature readings over five consecutive days. Results showed only a low correlation (r^2^ = 0.24) between orbital region and body temperature but produced a positive correlation between the orbital and anal regions (r^2^ = 0.43). Their findings differ from those reported by Athaíde et al. [61], who found a positive correlation (r^2^ = 0.65) of the maximum, median, and minimum temperatures of the lacrimal caruncle of eye and orbital region with the rectal temperatures of female Murrah buffaloes under two climatic conditions: with and without access to shade. Another study that included buffaloes (*Bubalus bubalis*) reported that ocular temperatures in the orbital region had a positive correlation (r^2^ = 0.92) with body temperatures in animals fed in open pasture [62].

In addition, it has been reported that the sensitivity and specificity of IRT with *Bos indicus* in an experimental model of febrile cows identified weak correlations between rectal and ocular temperatures in the orbital region (*regio orbitalis*) and readings from the muzzle in the nasal region (*regio nasalis*) and the lacrimal caruncle of the eye in the orbital region (*regio orbitalis*) (r^2^ = 0.38, 0.28, 0.27, respectively), with sensitivity values of 88, 90, and 82% for the respective regions, but low specificity that did not exceed 32% [63]. These findings suggest only limited usefulness of IRT in the orbital region during febrile states in this species.

According to the evidence presented, the thermal window of the lacrimal caruncle of the eye in the orbital region shows only a weak specific relation between thermal states and SyNS activity [64,65], thus raising doubts as to its reliability. Furthermore, diverse factors, both endogenous—sex, age, breed—and environmental—hour of the day, location—are components that could affect temperature evaluations and can lead to inadequate interpretations of readings taken in these body regions, as has been observed in other species, such as equines [66].

## 4. Nasal Region (*Regio Nasalis*)

**Figure 5:**
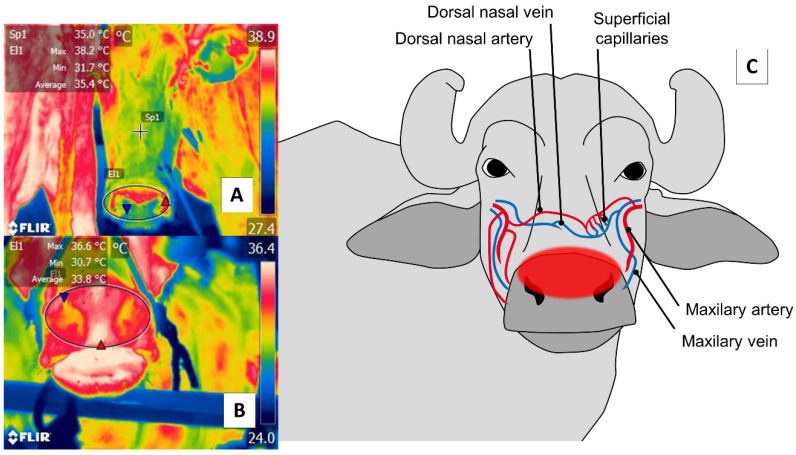
Nasal thermal window. (**A**) Cattle; (**B**) river buffalo. A circle is drawn (El1) around the muzzle of the nasal region (*regio nasalis*) to include both nostrils and the middle groove. This region permits evaluations of two conditions: first, changes in the microcirculation of the surface capillaries of the maxillary artery, as shown in (**C**); second, the elimination of water vapor during the respiratory process that makes it possible to assess, from a distance, the respiratory rate by observing changes in the thermal pattern at the central level of the nostrils.

With respect to the nasal region, detecting thermal changes in the regio naris of the muzzle, including both nostrils, responds to the blood irrigation provided by the maxillary artery and surface capillaries. This vasculature is schematized in Figure 5. Through this window, it is possible to evaluate the elimination of the vapor produced during the respiratory process to non-invasively determine thermal states and respiratory rates (RR) [2,12,67].

The first use of this thermal window was reported by Stewart et al. [2], who integrated IRT with accelerometers in a study of 22 cows (Friesian and Friesian × Jersey breeds) aged 5.1 years to evaluate RR and the flinch, step, kick response (FSK). The thermal response of the muzzle of the nasal region (*regio nasalis*) window reliably predicted heart rate variability due to changes in RR. Similar to these results, Lowe et al. [12] reported a high correlation (r^2^ = 0.92) upon comparing the conventional method of counting movements of the flank area during the respiratory cycle to thermal fluctuations around the nostrils using thermographic recording in five 27 ± 3.7 days Hereford’s calves. However, due to their size and age, these animals can be difficult to evaluate in terms of warming or the entrance of cool air during exhalation and inhalation, respectively.

Those changes in the vapor released at the moment of exhalations, and changes in the microvascular pattern of the region that can be detected in radiometric thermal images, justify using this thermal window to determine states of health—automatically and remotely—in extensive production systems, as is the case of most large ruminants, including the river buffalo [30]. For this species, the suggestion is to evaluate the nasal window and the pectoral regions (*regiones pectoris*) (ribs) as windows for linking temperature with efficiency and productive performance. According to a comparison of 75 buffaloes from three genetic groups (Jafarabadi, Mediterranean, Murrah), IRT made it possible to classify the animals into low, medium, and high-efficiency groups, depending on the temperature of specific body regions [68]. However, the usefulness of IRT for determining RR at a distance is not yet well established in this species, though it is for cattle.

As a result, the window of the nasal region (*regio nasalis and regio naris*) is currently considered only a potential option for estimating states of health and determining RR remotely in large ruminants. However, as occurs in human medicine, clinical applications are required to define the degree of sensitivity and specificity in different animal species [69].

## 5. Udder Region (*Regio Uberis*) and Mammary Gland

**Figure 6:**
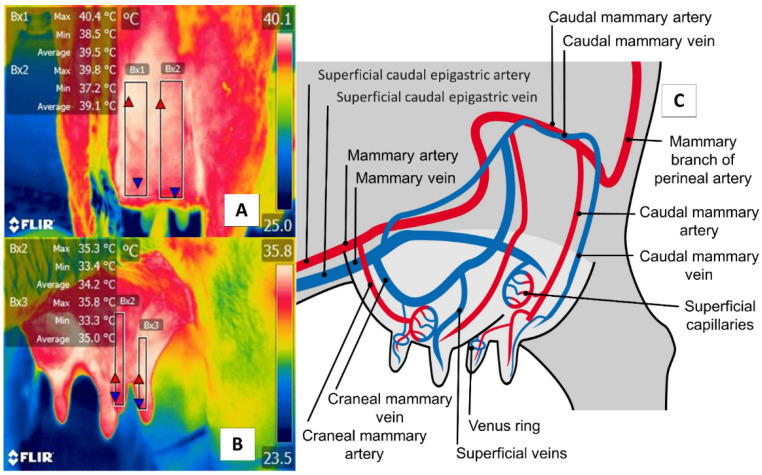
Thermal window of the udder region (*regio uberis*), including the mammary gland, in dairy bovines (*Bos*) and river buffaloes (*Bubalus bubalis*). (**A**) Dairy bovine (Holstein *Bos Indicus*, in production) are represented in the Figure with a square (Bx1) that extends proximally from the abdominal insertion of the mammary cistern and distally to the insertion of the teat, without considering the temperature of the latter. (**B**) River buffalo. The window is traced from the distal region of the teat to the cistern of the mammary gland. For this species, the full teat is considered, unlike in dairy bovines. Although significant anatomical differences exist between these two species, microcirculatory changes from the mammary arteries and veins can be obtained, as shown in (**C**). The justification for using this window in IRT is based on the fact that mastitis is characterized by bacterial colonization in the mammary gland generates a local inflammatory process due to the presence of proinflammatory cells that produce a secretion of prostaglandin, histamine, serotonin, and interleukins, which, in turn, trigger vasodilatation of the mammary capillaries, increasing the temperature of the region by 1.5 °C. This inflammatory increase could correlate with the rise of somatic cells [72].

The udder region (*regio uberis*), including the mammary gland, is another essential anatomical region, due to clinical conditions (e.g., mastitis) that have severe repercussions on production when cases become acute. As Figure 6 shows, the udder region (*regio uberis*) window, including the mammary gland, covers the body of mammary tissue to capture radiation emitted by the mammary artery and veins. Unlike in cattle, in river buffaloes, this approach also considers tissue at the teat level. Despite these anatomical differences, this thermal window has often been used to detect cases of subclinical mastitis [70,71].

For example, in an experimental model of induced mastitis in six bovines using IRT, Hovinen et al. [73] identified an increase of 1.5 °C in the temperature of the skin of the mammary gland, which they were able to associate with other signs of inflammation, such as increases in somatic cell counts and rectal temperatures.

Recent studies comparing the California mastitis test and somatic cell counts suggest that IRT is a highly sensitive tool for detecting mastitis. A study of 62 Brown Swiss cows, for instance, found a high positive correlation among IRT, scores on the California mastitis test (r^2^ = 0.86), and somatic cell counts (r^2^ = 0.73), with sensitivity and specificity that reached 88.9 and 98.9%, respectively [74]. In this case, river buffaloes show a similar tendency, according to observations by Sarubbi et al. [71], who reported a positive correlation (r^2^ = 0.64) between thermal changes in the mammary gland and somatic cell counts in 192 females with mastitis induced experimentally during lactation.

The studies mentioned emphasize the usefulness of IRT as a technique for the early detection and diagnosis of subclinical mastitis in both species. However, it is suggested that evaluations of infrared radiation in river buffaloes need to consider uncontrolled productive and climatic conditions because, in this species, environmental temperatures and changes in respiratory patterns can alter the thermal response of this window [28]. In addition, but from an anatomical perspective, differences between these species must be considered when examining IRT images since the river buffalo’s mammary tissue has more prominent suspensory ligaments and longer keratinized teats [30,75].

Some studies of river buffaloes have questioned the use of this region for detecting mastitis because, according to Machado et al. [70], radiometric thermal images of the left and right regions of the rear udder present higher correlations with somatic cell counts compared to anterior sections. In addition, their findings revealed a difference between evaluations of the posterior and anterior udder regions that reduce the reliability of this window.

However, the scientific evidence analyzed does reflect a clear consensus regarding the relation of thermal changes in the mammary gland to a local inflammatory response derived from clinical mastitis, which generates a temperature increase in the region. Despite this consensus, additional studies are required to determine the sensitivity of this window in both cattle and river buffaloes.

## 6. Perineal Region (*Regio Perinealis*)

**Figure 7:**
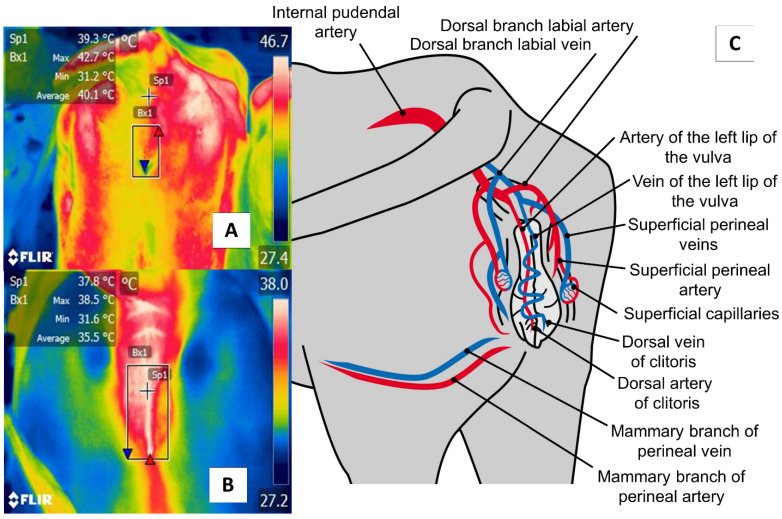
The perineal region (*regio perinealis*), especially vulvar thermal window, in dairy cattle (*Bos*) (**A**) and river buffaloes (*Bubalus bubalis*) (**B**). This window is marked by a square (Bx) placed from the coccygeal insertion of the vulva to the ventral commissure of this area, which permits taking readings of circulation from the internal pudendal artery (**C**). This artery emits capillaries in the vulvar labia that, during estrus, respond to increases in the concentrations of prostaglandin E2α that cause vasodilatation of the capillaries and, with this, greater temperature irradiation during this physiological stage that permits the detection of estrus in a non-invasive manner.

The perineal region (*regio* *perinealis*) is the surface area over the perineum and adjacent parts. In cows, this region is bounded dorsally by the tail root and ventrally by the attachment of the udder. The perineal region (*regio* *perinealis*) is divided into the anal (*regio analis*) and urogenital (*regio urogenitalis*) regions, including the external portions of the vulva [76]. The thermal window of the vulva frames the urogenital region (*regio urogenitalis*) in females. In this zone, the labia receive blood flow from the internal pudendal capillaries, as shown in Figure 7. These capillaries undergo dilatation during estrus that causes a temperature increase. For this reason, this anatomical region has been studied to determine its usefulness for detecting the onset of estrus [29,51].

Radigonda et al. [77] applied IRT to compare hormonal ovarian activity (by measuring estrogen and progesterone levels), animal breeding by artificial insemination, and changes in the thermal radiation of the vulvar window in 150 non-lactating Bradford cows. The authors found a significant difference be-tween the temperature of the animals that presented ovarian follicles and those that did not present ovarian activity (34.2 ± 1.8 °C and 35.4 ± 1.0 °C, respectively). They concluded that the temperature fluctuations detected by IRT could provide a support tool for detecting ovarian activity and reproductive states.

These findings were confirmed in a later study of 18 multiparous cows under a protocol of synchronized estrus and 18 pregnant cows used as a control group. That study evaluated IRT in various thermal windows as well as behavioral indicators of heat, while also verifying the condition of the ovary by ultrasonography. Results showed that the temperature of the perineal region (*regio perinealis*), especially the vulvar area, the orbital region (*region orbitalis*), including the eye, facial regions (*regiones* *faciei*) such as the cheek, neck regions (*regiones colli*), the wither region (*regio interscapularis*), the flank region (*regio abdominis lateralis*), and the rump region (*regio glutea*) all increased by approximately 1.2 °C and remained in that range for 24 and 48 h before ovulation, compared to the day of ovulation and four days before it. These data reveal the relation between temperature variability in this region and estrus in cattle [51].

Talukder et al. [29] evaluated the specificity of IRT in 30 female bovines that were close to ovulation, targeting various corporal regions (the eye in the orbital region (*region* *orbitalis)*, the ear in the auricular region (*regio auricularis*), the muzzle in the nasal region (*region nasalis*), and the vulva in the urogenital region (*regio urogenitalis*)), and taking two readings per day. Data on activity levels, rumination, progesterone concentrations in the milk, and recordings of insemination to estimate dates of ovulation were all collected to carry out a more integral evaluation. In that study, IRT of the vulva exhibited a higher temperature with a specificity of 80% but low sensitivity (21%) compared to the other indicators used, which determined sensitivity and specificity above 80%.

Studies of the river buffalo have reported a similar response, according to findings by Ruediger et al. [78], who evaluated temperature oscillations in the vulva and progesterone concentrations in 40 Murrah buffaloes in a synchronization protocol with progesterone. They observed that the temperature of the vulva increased during estrus and maintained an inversely proportional correlation to progesterone levels (r^2^ = 0.70); that is to say that progesterone levels increased to the degree that the temperature of the urogenital region (*regio urogenitalis*), especially the vulva, decreased. This thermal relation of the urogenital region and the estrus cycle is described as hyperthermia before ovulation that decreases later in that process [79]. However, the precision of this window, can be affected by environmental factors, such as solar radiation or wind, so it is essential to consider these elements when reporting final values, as other studies of river buffaloes also suggest [61]. Climatic changes and their link to reproductive parameters were analyzed by Yadav et al. [80] in 130 male Murrah buffaloes (breeding bulls). The scrotal region (*regio scrotalis*) temperature and increased dorsal-ventral temperature gradient of this zone in a cool climate were associated with better semen quality that translated into greater motility and higher sperm concentrations. This suggests that IRT can be used as a complementary method for andrological evaluations in this species [81].

In summary, the urogenital region (*regio urogenitalis*), especially the vulva, is a specific window that can aid in determining ovarian activity in large ruminants, where thermal behavior shows a temperature increase during the estrus cycle followed by an evident decrease during ovulation. However, the scant information available regarding this window and the diverse environmental and species-specific factors that intervene as elements of variability can generate alterations in the sensitivity and specificity of IRT. Therefore, these elements require thorough analyses to reach conclusions on the usefulness of this region in cattle and river buffaloes.

## 7. IRT and the Assessment of Pathological States

Initially, the application of IRT focused on evaluating inflammatory processes in specific regions, such as the hoof, where its use is important for the early detection of injuries and inflammatory pain that can impact production, feed intake, and animal welfare [82,83]. When animals suffer from laminitis, an increase in the skin temperature in the coronary band can be detected by IRT [84,85]. In this regard, a study carried out with 139 lactating dairy cows used IRT to evaluate the thermal response of the coronary band and the surrounding skin of the hoof and identify hoof lesions such as digital dermatitis and ulcers. The temperature in the wounded animals was 2 °C higher than in the healthy subjects. That study determined a sensitivity and specificity of 77.8% and 65.8%, respectively [86].

A similar range of specificity (85.7%) and sensitivity (82.9) was found by Alssaod and Büscher [87], who collected 626 images of three conditions: 24 dairy cows before and after hoof-trimming, healthy animals, and animals with some degree of lameness. The temperature of the coronary band and the skin had a significantly higher value in the sick cows; however, factors, such as ambient temperature and the temperature of the milking parlor, had a positive correlation (r^2^ = 0.92) with the trimming period temperatures. The aforementioned is noteworthy because it is essential to recognize the influence of the environment on skin surface temperatures, as discussed later.

Another suggested application of IRT in animals is to detect infectious and febrile states. During an infectious response, body temperature increases due to the presence of IL-1 and PGE2-alpha. This increase can be detected in thermal images [88]. A study that compared febrile and non-febrile cattle inoculated with *Escherichia coli* in the right hindquarter used this technology, complemented by various tools of geometric analysis (polygons, rectangles, and lines). It found that temperature increases greater than 2.06 °C were detected by the method suggested by the author [89].

Automated IRT can also assist in diagnosing complex respiratory disease in calves. In this line of research, Schaefer et al. [90] evaluated 65 dairy calves weighing ~220 kg, exposed to standard transport and industrial practices. IRT detected and associated higher temperatures (35.7 ± 0.35 °C) with clinically diagnosed respiratory diseases in the animals.

As these cases show, IRT is a valuable tool for preventing and detecting pathological states in domestic animals, due to the several inflammatory responses that increase body core and skin surface temperatures, which can be identified as infrared radiation [20,91,92]. Nonetheless, it is important to consider the influence of environmental factors on temperature readings taken from the animals evaluated to understand the results and then implement preventive strategies objectively.

## 8. Environmental Influence on the Physiological Responses of Thermoregulation

**Figure 8:**
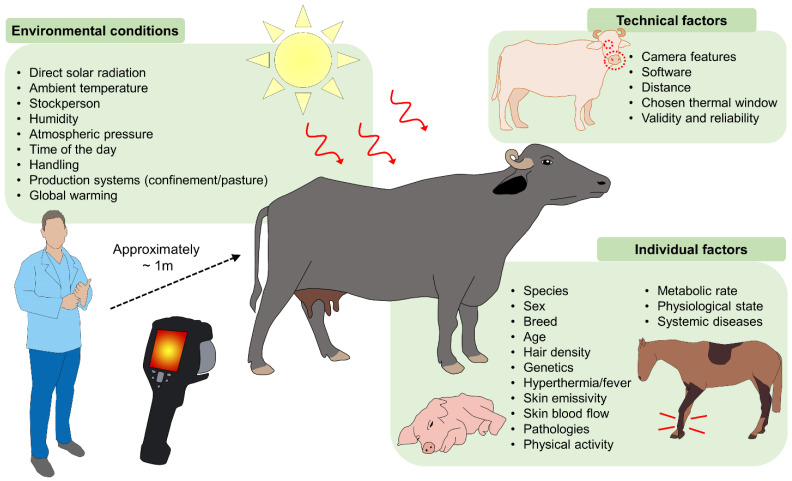
Environmental, technical, and individual factors that can influence the thermal responses of domestic animals. As a non-invasive tool that measures thermal fluctuations in the surface skin of animals, IRT can be affected by diverse agents, both extrinsic and intrinsic. Critical environmental conditions include direct solar radiation, ambient temperature, humidity, other climatic factors, and elements that depend on the stockperson, handling, and animal management. Individual factors that must be considered are species, breed, anatomical particularities, and acquired conditions, such as physical activity, systemic diseases, and physiological states. Potentially significant technical factors include the distance between observer and animal, the angle at which readings are taken, and the thermal window chosen.

The clinical utility of thermal windows and IRT for recognizing a painful event or predicting pathologies in large ruminants and other animals must consider all environmental conditions, as well as individual and technical factors that can alter evaluations and the accuracy of temperature readings [7,9,21,91] (Figure 8).

For humans, to give one example, there is a recommended room size (2 × 3 m^2^) to maintain a homologous temperature. The temperature recommended is 18–25 °C, depending on the region selected for the study. This range prevents physiological events (e.g., shivering or sweating) that occur under exposure to extreme temperatures. Applying formulas to calculate skin temperature, regardless of the ambient temperature and humidity, atmospheric pressure, and radiation is another factor to consider [92]. In terms of individual characteristics, pathological conditions, such as a bacterial infection in febrile pigs caused by *Actinobacillus pleuropneumoniae,* have shown a linear relation (r^2^ = 0.97) between ambient temperature and the mean body surface temperatures, where a 1 °C increase in the former caused a 0.40 °C increase in the body temperature of pigs in climates between 10 and 32 °C [93]. The thermal window chosen for that study was an area extending from the middle of the scapula to near the midpoint of the rear limb. Moreover, dorsoventrally from the shoulder to the olecranon and from the tuber coxa to the stifle. Another example of an individual factor is the strong correlation between ambient temperature and forelimb joint temperatures in racehorses, a parameter that could aid in the early detection of injuries or inflammation [94]. The correlation of ambient temperature has also been observed in dairy cows, where measurements of the surface temperature of the eye, ear, cheek, forehead, flank, rump, forelimbs, udder, and rear udder in dairy cows were affected and to be more sensitive to environmental conditions than rectal temperatures [95]. Ambient temperature, however, is not the only climatic factor that can affect IRT readings, for the presence of wind and direct solar radiation loading may have effects as well. In addition, technical factors, such as the camera-to-object distance and emissivity, can alter responses in diverse thermal windows (e.g., in the eye temperature of cattle) [6,7,14,48].

**Figure 9:**
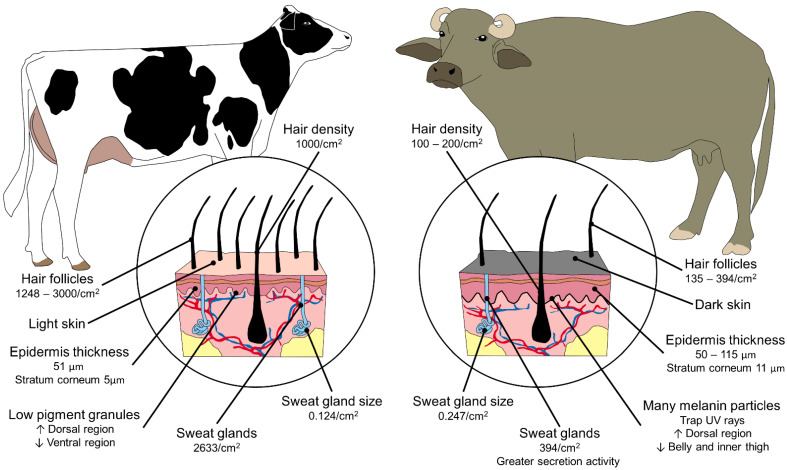
Morphological differences in skin characteristics between cattle (*Bos taurus* and *Bos indicus*) and river buffaloes (*Bubalus bubalis*). The skin and structures involved in the thermoregulation of buffaloes (number of hair follicles, hair density, epidermis thickness, melanin concentration, number and size of sweat glands) differ significantly from those in cattle, and influence the deficient thermoregulation mechanism of this species [104,105,106,107].

In the river buffalo, increases in the environmental temperature trigger physiological and behavioral responses (i.e., wallowing and muddying in water or seeking shade) when the peripheral and central thermoreceptors perceive changes in climatic conditions [96,97]. These behaviors serve to efficiently thermoregulate the animal through the vasodilatation of a large number of blood vessels in the skin [6,9,98,99]. However, buffaloes are particularly susceptible to heat stress especially when exposed to direct sunlight, due to their dark skin with high absorbance and transmittance and deficient dermal mechanisms for dissipating latent heat, [6,7,21,100,101,102]. This effect can also be seen in black and white cows that present greater absorption of solar radiation, though to a lesser degree than buffaloes during hot periods. However, in addition to color, coat thickness (and its transmittance), is another factor that can condition the amount of energy that is transferred along the fur and reaches the epidermis [6,7,9,103] (Figure 9).

**Figure 10:**
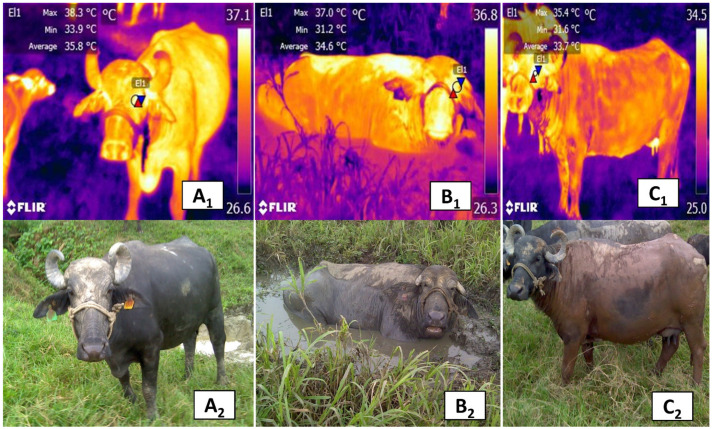
Thermal response of the orbital region of river buffaloes (*Bubalus bubalis*) under direct solar radiation, then during and after wallowing. (**A_1_**_,_**A_2_**) Effect of direct radiation. The temperature of the orbital region (El1) shows a maximum temperature of 38.3 °C (red triangle) and a minimum of 33.9 °C (blue triangle). These temperatures are due to the effect of heat dissipation through vasodilation of skin blood vessels. (**B_1_**,**B_2_**) Wallowing and its effect on orbital temperature. Decreases in the maximum and minimum ocular temperatures of 1.3 °C (red triangle) and 2.7 °C (blue triangle), are observed when the animals submerge in muddy waters to thermoregulate. (**C_1_**,**C_2_**) Effect of wind on orbital temperature after muddying. The maximum and minimum orbital temperatures decreased by 2.9 °C (red triangle) and 2.3 °C (blue triangle), compared to baseline values. This effect was caused by mud in the dorsal region and the combined cooling effect of convection of the wind and evaporation of water in mud on this zone; a strategy river buffaloes use to reach thermal comfort.

As a result, buffaloes must dissipate heat by lying in swampy areas or mud for long periods [21,99,108], especially during the hot season. Providing shelter and shade to buffaloes mitigates all the effects from heat stress, such as increased rectal temperature, respiratory rate, and plasma cortisol concentration [98]. The effects of shading also translate into greater weight gain than animals kept directly in the sun [109]. When exposed to direct solar radiation, other alterations observed in these animals are excessive salivation, which corresponds to a second phase panting with an increase in alveolar ventilation, a tendency towards respiratory alkalosis and hyperthermia and hyperthermia brought the inability to lose sufficient latent heat [110]. For example, a preliminary study of river buffaloes (*Bubalus bubalis*) found that wallowing and muddying are mechanisms that mediate thermoregulation in this species, as can be seen in the orbital region (*regio orbitalis*) of the animals shown in Figure 10.

This natural behavior of the water buffalo has additional benefits for these animals: protection against ectoparasites, and significant reductions of rectal temperature, water intake, and free triiodothyronine (an indicator of metabolic changes related to food intake and the influences of ambient temperature and humidity) [63,111,112,113]. In free-ranging buffaloes, feeding (including grazing and rumination) and resting represent the two main behaviors performed. In contrast, when animals are managed in intensive production systems, these natural behaviors are restricted, and the development of abnormal behaviors, such as aggression or sucking and allosucking, can reduce their welfare [114,115,116].

Despite scientific data indicating a direct influence of environmental factors on the physiological responses of these animals under high ambient temperatures [21], farmers often neglect this area and its negative consequences for their animals [117]. The neuroendocrine response to heat stress negatively affects behavior and productive and reproductive efficiency, with consequences for milk and meat production, growth, and fertility [104]. Moreover, heat stress has been shown to affect the gene expression of cytokines and their receptors, consequently affecting immune functions [118,119]. Hence, it is essential to acknowledge the influence of ambient temperature on the thermoregulation of the river buffalo, but also factors of this kind when measuring thermal and microvascular changes in animals. Perhaps an evaluation of different animals, but at the same time of day, would permit a more accurate interpretation.

## 9. Perspectives and Areas of Opportunity

Modifications of body temperature can result from pathological and physiological processes that are detectable in the thermal windows described above. The above opens a field of application that promotes the use of thermography for quantifying levels of thermal radiation in animals, not only during pathological disorders but also as a complementary tool for preventing and/or detecting diseases that are identifiable in animals, whether apparently healthy or ill, based on changes in surface microcirculation [20]. This suggests the need to analyze the potential usefulness of this tool in milking rooms, with calf feeders, and at watering stations [20], areas where radiometric thermal images can be obtained using portable devices or fixed cameras. Portable devices permit mobility and can be focused on a specific window but are limited in recording frequency. Fixed cameras installed in strategic places, in contrast, can evaluate in real-time for prolonged periods [120]. It is important to consider these characteristics and incorporate them adequately in future research since the IRT technique can be utilized to assist in the early detection and diagnosis of physiological alterations by comparing normal thermal parameters to even minimal temperature modifications.

The temperature responses of the body’s surface to vascular changes derived from multiple events also make it possible to determine patterns of thermoneutrality in animals and establish their levels of thermal comfort. This is a fundamental aspect in handling animals raised for production because heat stress causes a vasodilatory response that increases heat exchange with the environment and, as a result, surface temperature [95,118,119,121,122,123,124]. This alteration, which can be detected in thermal windows, impacts animals’ health, welfare, and productive and reproductive efficiency, so monitoring their condition utilizing the heat irradiated in specific body regions and measuring the temperatures, is a potential strategy for mitigating abnormalities in the fertility of both males and females, and for reducing associated pathologies, such as mastitis. However, additional research is required from the perspective of animal welfare to possibly validate the ocular window in the presence of nociceptive stimuli or stress [20,120].

Several advantages of IRT and the thermal windows discussed above have been postulated, especially non-invasiveness, real-time recording, and emissivity [20]. In the studies published to date, this tool has not only provided information on diseases and painful processes, but has also emerged as a predictor of meat quality by tracing animals from the farm of origin through the *post-mortem* evaluation [123]. In this case, by distinguishing autonomous modifications in blood flow towards the surface capillaries derived from a state of pre-slaughter stress, IRT can help prevent impacts on the organoleptic and physicochemical characteristics of meat, thus offering a resource that promotes the quality and innocuousness of foods in addition to its role as a predictor of poor animal welfare.

Future studies of the use of thermal windows, peripheral thermoregulation, and body heat production in cattle and the river buffalo should adopt a comparative focus to contemplate the anatomical and physiological differences between these two species [124]. The goals are to adopt these techniques in production units practically and achieve a more objective interpretation of the readings they provide. Future research should also focus on the following aspects: developing experimental designs to study environmental factors, specific characteristics of the animals (e.g., breed, sex, age, skin thickness, tissue disposition), and technical elements (e.g., the angle at which thermograms are taken, capture distance, etc.) [125]. All of these factors can help improve measurements of thermal changes in microcirculation to foment their evaluation during diverse functional processes in animals.

## 10. Limitations of Infrared Thermography due to Environmental Conditions

The main limitations of IRT are related to environmental conditions, such as solar radiation, which can potentially alter readings, as has been seen in humans [92]. A study carried out with dairy cows found that the velocity of the wind can also alter results, as speeds of 7 km/h decreased the temperature of the eye by 0.43 ± 0.13 °C, and at 12 km/h by 0.78 ± 0.33 °C. In animals with high sweating rates, when in places with larger wind velocities, the combined effects of convection and evaporation may lead to increased evaporation rates with consequent decreases in temperature at the sites of sweat evaporation, leading to some errors. In contrast, in animals kept under direct solar radiation, the orbital region temperature increased by 0.56 ± 0.36 °C [48]. In other species, such as the rabbit, reports show that the temperature of the orbital region—though it is a good region for evaluating stress—can be affected by relative humidity, ambient temperature, and ventilation, inducing variation in IRT readings [126]. Okada et al. [127] stated that in addition to the factors mentioned above, the distance to the object and the angle of the focal length used to obtain optimal thermograms may also place serious limitations on evaluation and must be taken into account by researchers.

## 11. Conclusions

In conclusion, it is evident that the anatomical and physiological differences among large ruminants and the impact of the environment, gender, and species, are elements that can influence the interpretation and validation of thermal windows, which may generate distinct responses in the same window. These factors must all be rigorously accounted for in future research designed to analyze the microvascular changes characteristic of each body region in order to achieve an integrated understanding of the thermoregulatory responses of different thermal windows and then implement this knowledge in clinical medicine for domestic bovines of the genus Bos and the river buffalo. When used with large ruminants, IRT gathers information from thermal windows that can assist in diagnosing specific pathologies and in determining the physiological states and welfare of animals. However, interpretations depend significantly on the region evaluated, the species studied, and environmental and technical factors that can affect our understanding.

At first, scientific findings on the orbital region (*regio orbitalis*) described the lacrimal caruncle of the eye as a zone whose vasomotor response was associated with a thermal change influenced by the peripheral activity of the SyNS during painful and pathological processes and stress. However, later research found that diverse endogenous factors, such as sex and breed and environmental components (hour of the day, location), can affect temperature evaluations and lead to in-adequate interpretations of the information recorded in this region. Meanwhile, the association of the nasal region (*region nasalis*) window with processes of exhalation and inhalation and the increase or decrease, respectively, of the temperature around the nostrils made it possible to use this window as an alternative for evaluating the respiratory function at a distance and correlating it with health, temperatures in other body regions, and productive parameters. Despite these advances, however, it is essential to consider such factors as climate, the age of the animals, and the need for additional clinical studies of large ruminants to determine degrees of sensitivity. In this regard, the udder region (*regio uberis*), especially in the mammary gland thermal window, shows high sensitivity and specificity of concordance among local inflammatory responses, vasodilatation of mammary capillaries, and the consequent temperature increases in tissues. As a result, this window has significant diagnostic value for detecting subclinical mastitis that is further sustained by high correlation levels with clinical studies, such as somatic cell counts and the California test. One aspect of this window that requires additional study involves the anatomical differences between the genera *Bos* and *Bubalus bubalis* (e.g., the latter’s prominent ligaments and longer teats, etc.) since these affect the delimitation and evaluation of the udder region and mammary thermography.

Finally, hyperthermia of the vulvar labia due to the contribution of the coccygeal capillaries, which is detectable through the urogenital region (*regio urogenitalis*) and vulvar window, can aid in verifying the stage of estrus in both species. Supporting findings from this window by measuring hormonal concentrations (e.g., progesterone) permits determining female reproductive states, though this zone is less sensitive than other thermal windows due to the effects of the environment, sun, and wind.
